# The Transformation of the Patient-Physician Relationship in Telemedicine: Sociological Approach in Dermatology

**DOI:** 10.2196/63591

**Published:** 2025-10-14

**Authors:** Edmond Démoulins, Dilara Trupia, Ludovic Martin, Alexandre Mathieu-Fritz

**Affiliations:** 1 Department of Dermatology, Angers University Hospital, 4 rue Larrey, Angers, 49100, France, 33 241353419; 2 Laboratoire Techniques, Territoires et Sociétés (UMR CNRS 8134), Paris, France; 3 Gustave Eiffel University, Paris, France

**Keywords:** telemedicine, ethics, sociology, patient-physician relationship, dehumanization, science, technology, and society

## Abstract

**Background:**

In 1999, the inaugural editorial of the *Journal of Medical Internet Research* underscored the imperative to evaluate the patient-physician relationship amid the advent of internet-driven medical practices. Even at that nascent stage, views diverged sharply. Some anticipated that novel communication technologies would enhance closeness between physicians and patients. Others feared these technologies signaled a degradation of interpersonal connections and a dehumanization of health care experiences.

**Objective:**

Reflecting on years of developments, we consider this an opportune moment to reassess transformations in patient-physician interactions, in which notions of distance and proximity are redefined in some unexpected ways.

**Methods:**

In this paper, we intend to focus on teleconsultation and tele-expertise, the two most commonly used services in our field, dermatology. To navigate these changes, a sociological perspective inspired by Isaac Joseph’s theoretical framework has been adopted. Joseph posits that service-oriented relationships depend on 3 core skill sets among participants: technical, civic, and contract management. In medicine, these translate to the clinical, social, and contractual aspects of care. Telemedicine programs introduce significant shifts and adaptations across these dimensions.

**Results:**

A primary challenge in telemedicine is the absence of comprehensive sensory information, a crucial component of traditional medical assessments. This article discusses how such technical alterations impact medical practice and necessitate a redistribution of roles. Telemedicine is not merely about adapting existing technical skills; it also demands new competencies, often referred to as “invisible work,” to make telemedicine work. Beyond its clinical dimensions, the therapeutic relationship is inherently social. It involves sending signals that confer respect and recognition of the other as a person. A common concern is that telemedicine might lead to reification, that is, turning human patients into objects to serve researchers and physicians, and although extensive literature suggests that physicians develop various strategies to mitigate this, other studies indicate an increased risk of reification in constrained contexts. Contract management skills consist of the parties involved reaching an agreement on the purpose of the service-related relationship and its practical applications. In telemedicine, this may involve reminding the patient that not everything can be done remotely.

**Conclusions:**

Through this analysis of the three components of the service relationship as defined by Joseph, we have highlighted the profound changes affecting the physician-patient relationship in the context of telemedicine. Dehumanization is an inherent risk in any medical health care partnership, regardless of whether patient care is delivered face-to-face or at a distance. While some studies have clearly documented the dehumanizing tendency of some telemedicine practices, telemedicine itself is neither reifying nor dehumanizing. The organizational arrangements, the context of work activities, the modalities of use in situ and the types of technological devices used are all contributing factors in defining the level of humanization in remote health care interactions.

## Introduction

The first *Journal of Medical Internet Research* editorial to appear in 1999 highlighted a need for assessment of the physician-patient relationship within the framework of the new medical practices generated by the internet [1]. Even then, opposing opinions had already emerged. Some claimed that new communication technology would bring physicians closer to their patients and widen access to care, while for others it represented a breakdown in interpersonal relationships, as well as a dehumanization of the health care experience [2-4]. These lines of argument have continued to evolve as technological advances have been made, particularly since the advent of telemedicine. The expansion of remote medical services (eg, teleconsultation, tele-expertise, and telemonitoring) has undoubtedly brought about one of the biggest transformations yet in the physician-patient relationship, yet human bodies have never been so physically far apart [5]. Very extensive literature has shone a light on these transformations by taking a balanced look at this world of medical services, in which the laws of distance and proximity have been rewritten in unexpected ways [6-9,9-14]. After several years of hindsight, we feel it is now timely to take a fresh look at the transformations that have taken place in physician-patient interaction in the specific case of teledermatology, and how the patient is viewed from a clinical perspective.

The dermatological field is a particularly interesting case study for considering the transformations in health care relationships associated with the development of telemedicine. First and foremost, dermatology is a highly clinical specialty that makes extensive use of practitioners’ senses (sight, touch, hearing, and smell) and requires physical auscultation of patients, sometimes from head to toe, in the clinical setting, as close as possible to their skin; in this respect, it is a particularly relevant indicator for assessing the effects of remote patient care in general. It is also a specialty in which telemedicine practices have become particularly widespread, especially since the COVID-19 pandemic, in two main forms: telemedicine visits and interprofessional consultations. A telemedicine visit—or *medical teleconsultation*—refers to a synchronous remote consultation between a physician and a patient. In contrast, an interprofessional consultation – or *tele-expertise* – involves an asynchronous request for a physician’s opinion, typically supported by a written report and/or clinical images of patients’ skin lesions.

There are several ways to study an interpersonal relationship, including psychological, sociological, and communication-based approaches. Given that the physician-patient relationship can be understood as a form of service relationship [15], we chose to adopt a sociological perspective inspired by the work of Isaac Joseph [15]. This perspective works on the premise that any service-related relationship is reliant on three main skill sets distributed among its participants: technical skills, civic skills, and contract management skills. With respect to medical practice, these skill sets refer to the medical/clinical, social, and contractual dimensions of the care relationship. First, technical skills refer to the practitioner’s clinical skills (including the use of the senses), medical knowledge, experience, and clinical reasoning used during an in-person consultation between a dermatologist and their patient. Technical skills are also used by the patient, including the ability to answer questions accurately and accept the clinical examination. Second, the civic skills described by Joseph are translated in the context of an in-person consultation through what we will call “social skills,” involving the recognition of the patient as a person in need of care, as well as recognition of the physician as the person qualified to provide that care. Lastly, contractual skills refer to the physician’s ability to respond to a patient’s request for care under appropriate conditions, and to the patient’s ability to accept this care.

With the introduction of telemedicine programs, each of these dimensions undergoes a wide range of shifts, adjustments, and transformations.

## Transformation of Technical Skills

The most obvious of the inherent limitations to the practice of telemedicine is without doubt deprivation of a whole set of sensory cues [16,17]. The term “cues” is used here in the sense of ecological perception: they constitute the material and practical elements required by individuals to reach their own judgment and formulate a response accordingly. In teleconsultation, for example, it is harder to see and hear each other. The physician is no longer able to touch or smell during clinical examination. Furthermore, the sensory work that is required to formulate a medical judgment also involves a constant assessment of the patient’s state of health, which extends beyond clinical examination and is carried out during the entire consultation through the sensory experience of the practitioner [18,19]. This embodied dimension of sensory work is profoundly modified in the context of telemedicine, where practitioners must develop new ways of reconstructing necessary information to formulate a diagnosis. It is therefore the technical dimension of the care relationship that is the most affected in telemedicine. There are a multitude of examples of this in dermatology, for the senses of (1) sight, (2) hearing, and (3) touch.

First, the sense of sight: dermatologists rely primarily on sight to make a diagnosis since dermatology is considered by the majority of practitioners to be a “visual specialty.” It is therefore reasonable to assume that dermatology is particularly compatible with the practice of remote medical services: visual perception is enabled through clinical images in tele-expertise and video transmission in teleconsultation. However, the way in which the camera is framed disregards a whole part of the patient’s body. It is common in clinical practice to encounter lesions that were not the primary reason for the patient’s visit. Telemedicine fragments this notion of global, holistic visual perception into images taken by a third party with whom the specialist does not necessarily share common knowledge or diagnostic skills [20]. Goodwin [21] defined ”professional vision” as the ability to transform a phenomenon into information that is relevant to a given professional practice. In teledermatology, this practice of professional vision has expanded to include numerous actors, experts and nonexperts alike, who may or may not be health care professionals. These actors may or may not know the patient’s clinical history or use connected (biomedical) equipment (eg, a dermoscope or smartphone), depending on the possibilities offered by the system, as well as their skill sets.

Second, the sense of hearing: in dermatology, this sense is used primarily during conversations with patients, which are generally perceived as being more fluid in face-to-face verbal exchanges. In teleconsultation, the considerable time lags in transmission that frequently occur make interaction seem more mechanical [8]. An increase in the sequencing of speaking time has been observed in telehealth services, with the effect of reducing interpersonal communication to an exchange of questions and answers. This mechanical style of communication may on occasion make it easier to get straight to the point [3,4]. In tele-expertise, these exchanges take place in written form using information systems that provide a more or less standardized wording framework.

Third, the sense of touch: a mucoid cyst on the fingers or toes might have the appearance of a highly suspicious purple nodule, but on physical examination is soft to the touch, thus reassuring the physician. Touch, whose function it is to confirm, reassure, and substantiate what has initially been identified visually by the physician, is obviously impossible in telemedicine. Palpation is, however, an essential part of reaching a diagnosis in certain cases. To replace touch, questions may be asked of the requesting physician in tele-expertise or of the patient in teleconsultation. Both need to find a way of transmitting haptic perceptions through a series of comparisons referring to common experiences [22].

Telemedicine produces profound changes in practitioners’ sensory work that go beyond simply adapting existing medical practices to new technologies [23]. The resulting limitations contribute in part to a greater sense of medical uncertainty, which can be offset by more detailed history-taking, by third party assistance with teleconsultation, and by use of specific technical devices such as digital biomedical equipment [24]. These compensation techniques produce shifts in the physician-patient relationship [25]. If, for example, a third party is required to direct the camera at lesions of interest, there is a shift from a 2-way relationship to a 3-way relationship that requires delegating certain technical tasks to a person who is in close physical proximity to the patient [7,26,27]. On other occasions, information that the physician seeks to obtain verbally may be supplemented by other contextual information gathered visually from the space used by the patient for teleconsultation, thereby intruding on the patient’s personal space [9,28].

There is far more to a physician’s technical skills than mere data collection. A phase of reflection is also required before providing the patient with explanations. The relocation of human bodies also has a profound impact on the way in which these phases unfold. In fact, every cognitive process involved in medical reasoning takes place in situ*,* within a physical environment where physicians contend not only with material conditions but also with their own bodies. Managing contextual factors such as fatigue, hunger, or variation in room temperature is a radically different experience in telemedicine. Some physicians practice tele-expertise outside of their usual workplace as well as outside of their working hours, sometimes carrying out other tasks at the same time [10]. These new methods of practice have yet to be thoroughly assessed. It is, however, important to treat any preconceived ideas about the shifts that have occurred in health care delivery with caution. A case in point is the way in which a physician in the middle of a night shift is able to contact a physician working in a different time zone. For example, emergency physicians already make use of interprofessional consultations (tele-expertise) by requesting medical opinions from teleradiologists [29].

Moreover, telemedicine is not merely an adaptation of existing technical skills; to make the system operational, it also requires the acquisition of new skills, often described as “invisible work” [27,30]. Notably, the broad range of knowledge and expertise required to tackle any problems related to telemedicine device malfunction (eg, problems with the internet connection, audio/video systems, and connection or use of biomedical equipment). Thus, technical skills not only refer to the transformations observed in the context of clinical activity, they also include all the skills required to operate technical devices in the extremely variable contexts of practitioners and patients, sometimes widening the digital divide, which has been extensively denounced in the literature on the uneven development of telemedicine [31].

## Rearrangement of Social Skills

Beyond its medical/clinical dimension, the patient-physician relationship is also a social relationship in its own right: we greet each other, we look at each other, we have conversations, and we listen to each other. We send signals to others as tokens of respect to indicate that they are recognized as a person, meaning that in this particular context they are also regarded not as a body-object, but as a patient-subject who is an active participant in their own health care [32]. This requires acknowledging patients’ individuality and taking into account their specific needs and choices in the clinical decision-making process to ensure that it is contextually appropriate. A concern that is frequently raised in the literature is that telemedicine tends to favor a certain reification of the patient, turning sentient beings into objects to serve research and health care [3,9,11,33,34]. Although objectification, translation of patients’ symptoms and subjective experiences into objective medical terminology, is a key part of the clinical approach to health care, this runs the risk of reducing the patient to a body, an organ, or an illness. Some authors call for caution regarding new technologies’ tendency to accentuate this process of reification of patients, whose identity is increasingly fragmented through the progressive digitization of their bodies into numerous bits of information, such as textual descriptions and iconographic representations that speak on their behalf [35,36]. This is indeed the case with tele-expertise, where the patient, or rather the tele-patient, is embodied by a set of textual and visual elements compiled and inscribed by another practitioner [23]. Hence only 92 of a random sample of 483 (19%) tele-expertise opinions we have given in dermatology at Angers University Hospital this year make mention of the patients themselves, instead focusing attention on the disease. For example, one of our conclusions contains the phrase “this is a case of erythema nodosum” instead of “this patient presented with erythema nodosum.”

However, a large body of literature indicates that physicians are developing a variety of strategies to compensate for these limitations and anticipated risks [23,30,37,38]. Physicians have therefore managed in some cases to use telemedicine services in ways that cultivate a health care partnership that is enriched when compared to that in a consulting room. Some patients seem to feel more at ease interacting with their physician from the comfort of their own home than in a physician’s office [8]. This type of interaction can even prove to be more informative, leading to a patient-physician relationship that is likely to result in greater medical benefits [12]. Enriched relationships are, however, far from the norm. In other fields, a greater tendency for reification has been observed when conditions are restricted [39]. One could extrapolate that with less time at their disposal, physicians are more inclined to be direct and to the point in telemedicine, focusing primarily on the disease, to the detriment of the patient. This also occurs in face-to-face patient care, but rearranging social conventions remotely has a greater tendency to favor reification [3]. Telemedicine is not dehumanizing in itself, but there is a risk of reification occurring if requirements for good practice are not met.

On the patient’s side, social skills must also be redefined. In a traditional medical consultation, the patient usually knows their physician, and a bond of trust is already established [40]. In telemedicine, due to the shift in usual reference points, such as location, timing, and modes of communication, this bond of trust must be reestablished [13,14]. This is even more true when the patients do not know the physician they are interacting with [14]. To establish and maintain this bond of trust between physician and patient in telemedicine, efforts and learning are required on both sides [13].

## Definition of New Contract Management Skills

The abovementioned technical and social shifts make a clear distinction between telemedicine and face-to-face health care. To make telemedicine work and to promote the development of a relationship of trust [38], it seems to be of particular importance to explain the difference to the patient. This explanation refers to contractual skills, one of the three components of Joseph’s [15] theory of the service relationship. Contract management skills consist of the parties involved reaching an agreement on the purpose of the service-related relationship and its practical application. In Joseph’s [15] theory, this contractual component influences the relationship between a physician and their patient. This relationship requires mutual agreement on the purpose and nature of the interaction—in this case, remote care. Accordingly, the representations that both the physician and the patient have of telemedicine and its effectiveness influence the relationship. Over the past 25 years, there has been an abundance of debate and prediction regarding the potentials of telemedicine and its use [2]. Two main advantages have been highlighted in particular: improved access to care and time savings for both health care professionals and patients.

Most often, telemedicine is presented as a solution to so-called medical desertification, which is especially the case in dermatology, where the shortage in France is unprecedented [41-43]. Initial attempts at using telemedicine to this end have opened up possibilities but have also revealed a number of limitations. Melanoma, for example, is relatively easy to diagnose using standardized images produced with a dermoscope [44]. However, an on-site primary care physician with access to a dermoscope is still required in medical deserts, as well as a surgeon to remove the lesion once the condition has been diagnosed. It is therefore important to remind patients that not everything can be managed remotely, and that telemedicine should alternate with face-to-face visits when needed [45,46]. Moreover, telemedicine is not accessible to everyone: several authors have shown that, rather than improving access to care, it can exacerbate health care disparities among vulnerable populations [47,48]. Only if the collective imagination finds ways of using telemedicine appropriately will patients take this information on board; for instance, by acknowledging that it enables the prioritization of the most urgent cases [13]. Conversely, promising that telemedicine will completely solve the issue of health care deserts may lead to false hopes and generate tensions within the physician-patient relationship.

It is also a common belief that by avoiding unnecessary trips, telemedicine intrinsically saves time for both the patient and the physician [10,42,49]. Yet maintaining top-quality telemedicine services demands a significant amount of time, in some cases possibly even more time than in face-to-face patient care [50]. We have also observed in our dermatology department that telemedicine services do indeed facilitate a rapid response to new appointment requests, namely by prioritizing patients who need to be seen urgently face-to-face. However, if this activity is not fully integrated into the organization of hospital work, the influx of new patients and the time required to respond to tele-expertise requests are likely to add even more to the existing workload. This has also been demonstrated in other medical contexts [51]. As an example of technological advancement that ultimately only adds to the workload, telemedicine is consistent with the social acceleration theory outlined by Rosa [52]. Telemedicine is not just a matter of conducting teleconsultations and responding to requests for tele-expertise remotely, it also necessitates the creation of a completely new system to manage patients who require face-to-face appointments following interprofessional consultations [53].

## Conclusion

Through this analysis of the three components of the service relationship as defined by Joseph [15], we have highlighted the profound changes affecting the physician-patient relationship in the context of telemedicine. The issue of transformation and the potential risk of dehumanization have been a matter of particular concern in telemedicine for the past 25 years. Yet this is an inherent risk in any health care partnership, regardless of whether patient care is delivered face-to-face or at a distance. While some studies have clearly documented the dehumanizing tendency of some telemedicine practices [3,4,9,11,33], telemedicine itself is neither reifying nor dehumanizing. In other words, compared to face-to-face care, in telemedicine, the patient is no more reduced to an object of care [30] and the clinical relationship is no colder or more limited to its purely technical aspects. The organizational arrangements, the context of work activity, the modalities of use in situ and the types of technological devices used are all contributing factors in defining the level of humanization in remote health care interactions [34]. It is also necessary to put into perspective the very idea of distance and proximity through telemedicine. In reality, the distancing of human bodies is a more general trend running through the history of modern medicine [54], which is increasingly based on laboratory medicine and imaging techniques [55,56]. While practitioners warn of the risks associated with this distancing, such as loss of auscultatory skills and the disintegration of the patient-physician relationship, research into the practical uses of telemedicine has revealed many compensatory efforts made by practitioners to ensure that telemedicine meets the technical, relational, and contractual requirements they deem necessary to carry out their work effectively [9,30]. In some cases, when these requirements are met, as paradoxical as it seems, distance can even bring health care actors closer together [6,12].

In any event, time is one of the main factors in the integration of telemedicine. While telemedicine took its first steps as long as 50 years ago, it became widespread on a massive scale within a matter of months after the lockdown of the general population in response to the COVID-19 pandemic [4,9]. However, appropriation of telemedicine services is impossible on such a rapid scale. Information technology/internet appropriation took several years and even several decades in the case of some physicians [57]. Acculturation to telemedicine is in its infancy. It is our duty to help it on its way by providing future generations of physicians with training and information by staying loyal to a noble vision and by establishing and upholding safeguards over the course of its development.

## Abbreviations

IC: interprofessional consultations
TV: telemedicine visits
